# Consolidation chemotherapy may improve pathological complete response for locally advanced rectal cancer after neoadjuvant chemoradiotherapy: a retrospective study

**DOI:** 10.7717/peerj.9513

**Published:** 2020-07-07

**Authors:** Jin Cui, Xue Dou, Yanlai Sun, Jinbo Yue

**Affiliations:** 1 Department of Graduate, Shandong First Medical University and Shandong Academy of Medical Sciences, Jinan, Shandong, China; 2 Department of Radiation Oncology, Shandong Cancer Hospital and Institute, Shandong First Medical University and Shandong Academy of Medical Sciences, Jinan, Shandong, China; 3 Department of Surgical Oncology, Shandong Cancer Hospital and Institute, Shandong First Medical University and Shandong Academy of Medical Sciences, Jinan, Shandong, China

**Keywords:** Colorectal cancer, Consolidation chemotherapy, pCR, Tumor downstaging, Neoadjuvant chemoradiotherapy, TME

## Abstract

**Background:**

Patients with locally advanced rectal cancer (LARC) have an improved prognosis if achieved a pathological complete response (pCR) on account of neoadjuvant chemoradiation therapy (nCRT). However, the proportion of patients achieving pCR is only 8–24%. The purpose of this study was to explore whether the addition of consolidation chemotherapy to nCRT could improve pCR rate in patients with LARC.

**Materials and Methods:**

The subjects were 144 individuals with clinical stage II (T3–4, N0) or III (any T, N1–2) LARC who had received neoadjuvant CRT followed by total mesorectal excision (TME). Eighty-three patients in the consolidation chemotherapy group received two cycles XELOX between CRT and TME, while 61 patients in the standard treatment group without consolidation chemotherapy. The pCR (ypT0N0), tumor downstaging (ypT0-2N0) after TME and adverse events (AEs) during and post treatment were compared between the treatment groups using multivariable logistic regression analysis. To adjust the unbalanced variables for the primary endpoint, logistic regression analysis and stratified analysis were performed.

**Results:**

The consolidation chemotherapy group improved pCR rate (19.3% vs 4.9%, *p* = 0.01) and tumor downstaging rate (45.8% vs 24.6%, *p* = 0.009) compared to the standard treatment group. After adjustment for clinical tumor stage, clinical nodal stage and time interval to surgery, patients with consolidation chemotherapy were more likely to reach pCR (adjusted odds ratio 4.91, 95% CI [1.01–23.79], *p* = 0.048). AEs during and post treatment in the two groups were 54.1% vs 49.3% (*p* = 0.57), respectively. In addition, the incidence of any grade 1–2 AEs in the two groups was 93.4% vs 95.1% (*p* = 0.93), while the incidence of grade 3 AEs was 1.6% versus 2.4% (*p* = 0.74), respectively. No grade 4 AEs occurred in two groups.

**Conclusions:**

The addition of neoadjuvant consolidation chemotherapy after CRT significantly increased the pCR rate and did not increase the AEs during and post treatment and in patients with LARC.

## Introduction

The current standard therapy for patients with locally advanced rectal cancer (LARC) is neoadjuvant chemoradiation (CRT) followed by total mesorectal excision (TME) and postoperative adjuvant chemotherapy (Macfarlane, Ryall & Heald, 1993; Seegenschmiedt, 2004). This trimodal therapy provides excellent local tumor control and long-term survival (Ludmir et al., 2017; Roh et al., 2009). However, TME is associated with some fatality, morbidity and long-term sequelae, with negative effects on quality of life. The pathological complete response (pCR), defined as the complete absence of tumor cells in the resected specimen and lymph nodes (ypT0N0) is associated with improved local control, overall survival, and disease-free survival which on behalf of improved prognosis (Kerr, Norton & Glynne-Jones, 2008; Maas et al., 2010; Zorcolo et al., 2012). Patients who have a complete response could be eligible for less invasive surgeries or even a watch-and-wait approach, questioning the added value of TME in these patients (Habr-Gama et al., 2004; Smith et al., 2012). However, the proportion of patients who achieve a pCR remains low with only 8–24% after neoadjuvant CRT (Maas et al., 2010). Increasing the rate of pCR, and therefore expanding the number of rectal cancer patients who could potentially benefit from a watch-and-wait strategy, has been a hot area of research for years (Smith et al., 2015). The intensified strategy includes escalating radiation dose, adding neoadjuvant treatment with induction or consolidation chemotherapy, and prolong the time of between neoadjuvant CRT and resection (Roh et al., 2009). However, the effects of these approaches on tumor response and long-term survival are still controversial. Two prospective phase II trials demonstrated that adding modified FOLFOX6 or XELOX after CRT and before TME increased pCR rate without increase the surgical difficulty in patients with LARC (Garcia-Aguilar et al., 2015).

The Timing of Rectal Cancer Response to CRT trial was designed to investigate the effect of adding an increasing number of cycles mFOLFOX6 (folinic acid, fluorouracil and oxaliplatin) after CRT and lengthening the CRT-to-resection interval on the rate of pCR in patients with LARC. The results showed that adding consolidation chemotherapy after CRT and delaying surgery increased the pCR rate without increasing surgical complications (Garcia-Aguilar et al., 2015; Marco et al., 2018). Kim et al. (2018) found that pCR and DS rate could be marginally improved with two cycles XELOX consolidation chemotherapy after preoperative CRT before TME. Will the above phase II trials result be the same in the real-world? Therefore, we aimed to investigate the effect of adding consolidation chemotherapy between CRT and resection on the pCR as well as surgical outcomes.

## Materials and Methods

### Patients

This study was a single-center retrospective cohort study based on patients with clinical stage II (T3–4, N0) or III (any T, N1–2) LARC who underwent neoadjuvant CRT followed by surgery at Shandong Cancer Hospital, affiliated with Shandong University, from January 2014 to December 2019. Eligibility criteria included a diagnosis of LARC with confirmed by pathological diagnosis; age was ≥18 years; invasive rectal adenocarcinoma within 12 cm from the anal verge; no history of receiving chemotherapy or pelvic radiation for rectal cancer; Eastern Cooperative Oncology Group (ECOG) performance status (PS) ≤2; no distant metastasis. The exclusion criteria were as follows: existence of distant metastases, concurrently diagnosed colon cancer, history of other cancer, other serious life conditions. Patients with a history of pelvic radiation, recurrent rectal cancer, metastatic disease, other primary tumors within the previous 5 years were excluded. The clinical T and N staging was using MRI scans according to the American Joint Committee on Cancer’s (AJCC) seventh staging system. Imaging studies including abdomen and pelvis CT, and rectal MRI were applied before treatments to assess primary tumor and exclude distant metastases. The study was approved by the Institutional Research Ethics Committee of Shandong Cancer Hospital in Jinan, Shandong (approval number #2019-23). The requirement for informed consent was waived because of the retrospective nature of the study. All methods were performed following the relevant guidelines and regulations.

### Treatment strategy

Each patient received initially radiotherapy (DT 50 Gy/25 fractions) concurrent with capecitabine (825 mg/m^2^ twice daily for 5 days/week during CRT). The choice of whether to consolidation chemotherapy was according to the physician’s decision. For patients without consolidation chemotherapy (group neoadjuvant chemoradiation therapy (nCRT)), TME was treated 4–8 weeks after the completion of CRT. For patients with consolidation chemotherapy (group CCT), two cycles of consolidation chemotherapy in XELOX (Oxaliplatin 130 mg/m^2^ day 1, Capectiabine 1,000 mg/m^2^ twice daily days 1–14 every 3 weeks) was administered between CRT and TME. Repeat every 3 weeks to a total of two cycles.

### Outcomes

We used the pCR rate to evaluate the efficacy of adding consolidation chemotherapy. The pCR was defined as the complete absence of tumor cells in the resected specimen and lymph nodes (ypT0N0). The tumor downstaging (DS) was defined as the proportion of patients with ypT0-2N0M0 (ypStage 0 or I) for those who underwent TME and assessed using the AJCC 7th staging system. In this study, we reported pCR and DS rate for outcome results. Besides, adverse events (AEs) during and post treatment were also compared between the treatment groups to evaluate the safety of consolidation chemotherapy.

### Statistical methods

All categorical variables were assessed using the one-sided Fisher’s exact test or Chi-squared test. Continuous variables were tested using the Student’s *t-*test. To adjust the unbalanced variables for the primary endpoint, logistic regression analysis and stratified analysis were performed, and one-sided *P*-value was derived. *P* < 0.05 was regarded as statistically significant in two-tailed tests. IBM SPSS STATISTICS version 25.0 (SPSS Inc., Chicago, IL, USA) was used for statistical analysis.

## Results

### Patient characteristics

Totally 144 eligible LARC patients with CRT were treated in our cancer institute from January 2014 to December 2019 were retrospectively reviewed. Eighty-three (58%) and 61 (42%) patients were categorized into with the consolidation chemotherapy group and without consolidation chemotherapy group respectively. The median age was 54 years (range 20–78). All of the patients were adenocarcinoma. A summary of the baseline for the 144 patients’ characteristics like the patient demographics and tumor characteristics of the two groups is presented in Table 1. No differences were found in demographics, tumor characteristics, or clinical and pathological features between two groups, indicating balanced groups in our study.

**Table 1:** Baseline for the 144 patients’ characteristics in consolidation chemotherapy (CCT) and neoadjuvant chemoradiotherapy (nCRT).

	nCRT (*n* = 61)	CCT (*n* = 83)	*P* value
Age (years)			
Median (range)	56 (24–78)	54 (20–72)	0.34
Gender			
Male	34 55.7%	57 68.6%	0.11
Female	27 44.3%	26 31.4%	
BMI (kg/m^2^)			
Median (range)	23.5 (17.3–30.4)	24.2 (18.4–33.9)	0.34
ECOG performance status			
0	28 45.9%	37 44.5%	0.87
1	33 54.1%	46 55.5%	
Clinical T stage			
T3	17 27.8%	33 39.7%	0.21
T4	44 72.2%	50 60.3%	
Clinical N stage			
N0	12 19.6%	12 14.4%	0.48
N1	38 62.2%	50 60.2%	
N2	11 18.2%	21 25.4%	
Clinical stage			
II	12 19.7%	12 14.5%	0.41
III	49 80.3%	71 85.5%	
CEA (ng/ml)			
Median (range)	4.3(0.93-152.2)	6.23(0.24-247.6)	0.32
<5 ng/ml	33 54.1%	38 45.7%	
≥5 ng/ml	28 45.9%	45 54.3%	

**Note:**

BMI, body mass index; ECOG, Eastern Cooperative Oncology Group; CEA, carcinoembryonic antigen.

### Surgical procedures and pathologic outcome

All of the patients eventually underwent TME with R0 resection. As shown in Table 2, there was no significant difference in the estimated blood loss and operation time during surgery. In the postoperative pathological staging, group nCRT had a significantly higher percentage of ypT4 than group CCT (63.9% vs 28.9%). The pCR rate in the CCT group was significantly higher than in the nCRT (19.3% vs 4.9%, *P* = 0.01). Also, DS rate (45.8% vs 24.6%, *P* = 0.009) was significantly higher in the CCT group than in the nCRT group. When analyzing time interval as a categorical variable, the highest proportion of patients in the nCRT group underwent TME within 6 weeks (60.6%) or between 6 and 10 weeks (32.7%) while in the CCT group most patients had TME between 6 and 10 weeks (61.4%) or after 10 weeks (27.7%) (*P* < 0.001).

**Table 2:** Differences in pathology outcomes between consolidation chemotherapy (CCT) and neoadjuvant chemoradiotherapy (nCRT).

	nCRT (*n* = 61)	CCT (*n* = 83)	*P* value
Interval time (weeks)			
Median (range)	5 (3–14)	9 (2–16)	<0.001
<6	37 60.6%	9* 10.9%	
6–10	20 32.7%	51 61.4%	
>10	4 6.7%	23 27.7%	
Approach			
Laparoscopic	18 29.6%	40 48.1%	0.02
Open	43 70.4%	43 51.9%	
Operation mode**			
APR	31 50.8%	34 40.9%	0.16
LAR	20 32.7%	40 48.1%	
Hartmann resection	10 16.5%	9 11%	
Operation time (min)			
Median (range)	215 (90–420)	210 (120–480)	0.53
Estimtated blood loss (g)			
Median (range)	50 (20–100)	50 (20–500)	0.23
ypStage			
0	3 4.9%	16 19.4%	0.008
I	12 19.7%	25 30.1%	
II	25 41%	28 33.7%	
III	21 34.4%	14 16.8%	
ypTstage			
0	3 4.9%	16 19.2%	0.001
1	3 4.9%	3 11%	
2	11 18%	29 27.7%	
3	5 8.3%	11 13.2%	
4	39 63.9%	24 28.9%	
ypNstage			
0	40 65.5%	69 83.1%	0.11
1	12 19.6%	8 9.6%	
2	9 5.9%	6 7.3%	
pCR rate	3 4.9%	16 19.3%	0.01
DS rate (ypStage 0 + I)	15 24.6%	38 45.8%	0.009

**Notes:**

* Nine patients had only one cycle of the XELOX because they refused the second cycle of XELOX.

** Patients with T4b tumors with adjacent organ invasion underwent combined resection.

APR, abdominoperineal resection; LAR, low anterior resection; pCR, pathologic complete response; DS, downstaging.

In stratified analysis, CCT was associated with a higher pCR rate in patients with a cT4, cN1 and 6–10 weeks of time interval (Table 3).

**Table 3:** Stratified analysis of the association between consolidation chemotherapy (CCT) or neoadjuvant chemoradiotherapy (nCRT) and pathological complete response (pCR) by clinical tumor stage, clinical nodal stage and categories of time interval.

Strata	pCR in CCT	pCR in nCRT	*P*-value
Clinical tumor stage			
cT3	2/17	4/32	0.09
cT4	1/44	12/50	0.002
Clinical nodal stage			
cN0	2/12	3/12	0.61
cN1	1/38	10/50	0.01
cN2	0/11	3/21	0.18
Time interval to surgery			
<6 weeks	2/35	0/9	0.46
6–10 weeks	0/21	11/51	0.02
>10 weeks	1/5	5/23	0.93

In multivariable analysis, the probability of pCR was significantly higher in the CCT group compared to the nCRT group (Odds Ratio = 4.91, 95% CI [1.01–23.79], *P* = 0.048 adjusted for gender, categories of CEA, cT-stage, cN-stage and categories of time interval) (Table 4).

**Table 4:** Multivariable analysis of the association between consolidation chemotherapy (CCT) or neoadjuvant chemoradiotherapy (nCRT) and pathological complete response (pCR).

	Crude OR (95% CI)	Adjusted OR (95% CI)	*P* value
Neoadjuvant therapy			
CRT	Ref.	Ref.	
CCRT	4.6 [1.3–16.6]	4.91 [1.01–23.79]	0.048
Sex			
Male	Ref.	Ref.	
Female	0.47 [0.18–1.25]	2.85 [0.93–8.75]	0.07
CEA			
<5 (ng/ml)	Ref.	Ref.	
>5 (ng/ml)	0.94 [0.35–2.52]	0.88 [0.28–2.67]	0.82
Clinical T-stage			
cT3	Ref.	Ref.	
cT4	1.21 [0.44–3.31]	1.38 [0.44–4.33]	0.58
Clinical N-stage			
cN0	Ref.	Ref.	
cN1	0.54 [0.17–1.75]	0.49 [0.12–2.01]	0.32
cN2	0.39 [0.08–1.84]	0.23 [0.38–1.42]	0.11
Time interval			
<6 weeks	Ref.	Ref.	
6–10 weeks	3.79 [0.79–17.97]	1.97 [0.30–12.9]	0.47
>10 weeks	5.72 [1.07–30.77]	4.41 [0.58–33.2]	0.14

**Note:**

CEA, carcinoembryonic antigen; OR, odds ratio.

### Safety

Table 5 describes AEs during the two groups. Nausea, diarrhea, anal pain, leukopenia and neutropenia were more common in CCT group than in nCRT group, whereas thrombocytopenia and anorexia were more common in group nCRT. Any grade 1–2 AE occurred in 57 of 61 patients (93.4%) in group nCRT and 79 of 83 (95.1%) in group CCT. Any grade 3 AE occurred in 1 of 61 patients (1.6%) in group nCRT and 2 of 83 (2.4%) in group CCT. No grade 4 AE was reported during treatment in both groups.

**Table 5:** Differences in adverse events (AEs) between consolidation chemotherapy (CCT) and neoadjuvant chemoradiotherapy (nCRT).

	nCRT (*n* = 61)		CCT (*n* = 83)	
	Grade 1–2	Grade 3–4	Grade 1–2	Grade 3–4
Any toxicities	57 93.4%	1 1.6%	79 95.1%	2 2.4%
Nausea	6	0	14	0
Fatigue	4	0	0	0
Diarrhea	5	0	21	0
Anal pain	4	0	17	1
Peripheral neuropathy	0	0	2	0
Anorexia	7	0	5	0
Abdominal pain	5	0	9	0
Constipation	2	0	3	0
Urinary tract infection	1	0	1	0
Myalgia	1	0	1	0
Sepsis	0	0	0	0
Leukopenia	8	1	26	2
Hand foot syndrome	0	0	2	0
Neutropenia	6	0	10	0
Anemia	2	0	3	0
Thrombocytopenia	3	0	0	0
Hyponatremia	0	0	1	0

As for surgical complications, there was no significant difference between postoperative morbidities in the two groups (Table 6). Incisional pain was the most common morbidity in both groups (21.3% and 18,1%). Surgical-site infection was more common in group nCRT than group CCT (14.7% vs 7.2%).

**Table 6:** The comparison of postoperative complications between the consolidation chemotherapy (CCT) and neoadjuvant chemoradiotherapy (nCRT).

	nCRT (*n* = 61)	CCT (*n* = 83)
Overall operative morbidity: *n* (%)	33 (54.1)	41 (49.3)
Intestinal obstruction: *n* (%)	0 (0)	0 (0)
Surgical-site infection: *n* (%)	9 (14.7)	6 (7.2)
Transient urinary dysfunction: *n* (%)	0 (0)	0 (0)
Pneumonitis: *n* (%)	1 (1.6)	3 (3.6)
Perineal wound infection: *n* (%)	2 (3.2)	7 (8.4)
Intraabdominal abscess: *n* (%)	3 (4.8)	7 (8.4)
Enteritis: *n* (%)	1 (1.6)	0 (0)
Catheter infection: *n* (%)	1 (1.6)	2 2.4
Anastomotic leakage: *n* (%)	3 (4.8)	1 (1.2)
Incisional pain: *n* (%)	13 (21.3)	15 (18.1)

## Discussion

How to increase pCR rate after neoadjuvant therapy and therefore to expand the number of LARC patients who could potentially benefit from a watch-and-wait strategy has been a hot research. In our study, we retrospectively used real-world data to demonstrate that the consolidation chemotherapy significantly increased the pCR in patients with LARC. Just as importantly, we showed that this approach appears to be safe from oncology and surgical perspective. It does not increase the risk of AEs or surgical complications before and after treatment. If these findings can be applied clinically, more patients with LARC will be eligible for organ preservation, which will avoid surgical sequelae and improve quality of life.

Currently, some prospective phase II trials had focused on the efficacy of consolidation chemotherapy between neoadjuvant CRT and TME in patients with LARC. In a nonrandomized phase II clinical trial from Garcia-Aguilar et al. (2015), patients received two, four, or six cycles of modified FOLFOX between neoadjuvant CRT and resection at different interval time of 12, 16, and 20 weeks, which resulted in improved pCR of 25%, 30% and 38% respectively compared with the pCR of 18% for patients without consolidation chemotherapy. In our study, the pCR rate was increased to 19.3% in the group CCT compared with the pCR of 4.9% for patients without consolidation chemotherapy. The pCR rate was 4.2% in our study for patients without consolidation chemotherapy, which is smaller than the data with 8–24% reported before (Van der Valk et al., 2018). Although the pCR rate in this study was lower than reported in both groups (4.9% and 14.3%), consolidation chemotherapy was related to enhanced tumor regression. The pCR rates in our study are lower due to that the patients in our study are unusually adverse in that 65% are cT4 which normally is associated with a low chance of achieving a pCR (Kim et al., 2018). Improved pCR and downstaging rates could be the result of the study treatment, but we cannot exclude that it may also have been affected by a significant delay of surgery in group CCT compared with group nCRT. Results from several retrospective studies have confirmed that longer chemoradiotherapy to surgery intervals are associated with a higher percentage of patients achieving a pathologic complete response (Calvo et al., 2014; Francois et al., 1999; Habr-Gama et al., 2004; Kalady et al., 2015; Tulchinsky et al., 2008; Moore et al., 2004; Sloothaak et al., 2013; Wolthuis et al., 2012; Zeng et al., 2014). In the Lyon R90-01 study (Francois et al., 1999), Francois and his colleagues found that 14% of patients who underwent surgery after 8 weeks of radiotherapy achieved a pCR, while only 7% of patients who underwent surgery after 4 weeks of radiotherapy achieved a pCR. However, considering the results of a large observational study that showed a 2–4% increase in pCR rates for each 1-week delay after the start of CRT within 17 weeks (Sloothaak et al., 2013), it is possible to explain the 14% increase in pCR rates for a delay of 2 weeks. The purpose of our study was to consider whether the addition of consolidation chemotherapy could increase the proportion of pCR patients regardless of the time interval. Inevitably, in a subset of patients, the addition of consolidation chemotherapy between neoadjuvant chemoradiotherapy and TME resulted in an unintended increase in the time interval. Therefore, we used stratified analysis to exclude the time interval difference between the two groups. The stratified analysis results show that CCT was associated with a higher pCR rate in patients with 6–10 weeks of time interval (Table 3). Also, the multivariable analysis which used logistic regression also suggested that the probability of pCR patients was significantly higher in the group CCT compared to the standard treatment group (Odds Ratio = 4.91, 95% CI [1.01– 23.79], *P* = 0.048 adjusted for cT-stage, cN-stage and categories of time interval) (Table 4). In a randomized phase II clinical trial, Kim et al. (2018) implemented a randomized phase II study, which suggested pCR and DS rate could be marginally improved with two cycles XELOX consolidation chemotherapy after preoperative CRT before TME. The interval time between the two groups were both 6–10 weeks but included a difference of only 10 days. Unlike our results, in their study, there was a numerical difference in pCR rates between the two groups, but it was not statistically significant (13.6% vs 5.8%, *P* = 0.167). However, the authors did not conduct further analysis to explore whether the difference in PCR was caused by the addition of consolidation chemotherapy or the extension of interval time.

As time prolongs, the potential development of pelvic fibrosis that could increase postoperative complications (Du et al., 2018). In the current study, patients’ operation time, intraoperative blood loss and postoperative complications were also collected. As shown in the Tables 2 and 3, there was no significant difference in operation time (*P* = 0.53) or intraoperative blood loss (*P* = 0.23) between the two groups. For surgical complications, overall surgical complications in group nCRT were slightly higher than those in group CCT (54.1% vs 49.3%, *P* = 0.57), the most common postoperative complication in both groups was incisional pain, and the incidence of surgical-site infection in group nCRT was slightly higher than that in group CCT.

Adverse events are an important part of evaluating experimental safety, so not only we reported surgical-related complications, we evaluated the incidence of AEs during chemoradiotherapy. In our study, the top three AEs were Leukopenia, Anorexia and Nausea in group nCRT, while Leukopenia, Diarrhea and Anal pain were in group CCT. The incidence of any grade 1–2 AE in our study was 93.4% in group nCRT and 95.1% in group CCT (*P* = 0.93), which was slightly higher than the previous randomized controlled trial (91% vs 92%). Considering the fact that the dose of oxaliplatin and capecitabine in our trial (100 mg/m^2^ and 850 mg/m^2^, respectively) was higher than in the previous trial (130 mg/m^2^ and 1,000 mg/m^2^, respectively), the incidence of AEs was similar to the previous trial which suggested that consolidation chemotherapy is a safe way to improve the proportion of pCR for patients with LARC.

Our study has several limitations. First, the use of a retrospective database limited our capacity to investigate other sources of potential bias. Second, the number of cases is limited, and it needs expanding the number of patients to confirm our conclusions. Approximately 14% increment in the DS rate and 21% increment in the pCR rate could be significant, but the sample size was small eventually may permitted a room for type-I and type-II error.

Up to now, modify and optimize the radiotherapy dose, chemotherapy regimen, treatment duration and interval time is still needed to seek the optimal treatment mode in patients with LARC. Nevertheless, the current study based real-world data demonstrated the role of consolidation chemotherapy may improve pCR rate after neoadjuvant CRT, adding a real-world data piece of evidence to support the effectiveness of consolidation chemotherapy in patients with LARC. Further randomized phase III trial is warranted to explore the role of consolidation chemotherapy.

## Conclusions

In conclusion, our study demonstrated that adding neoadjuvant consolidation chemotherapy after CRT significantly increased the pCR rate and did not increase the AEs or surgical complications in patients with LARC.

## Supplemental Information

10.7717/peerj.9513/supp-1 Supplemental Information 1 Raw data.

## Additional Information and Declarations

### Data Availability

The following information was supplied regarding data availability: The raw data are available as a Supplemental File.

## References

[ref-1] Calvo FA, Morillo V, Santos M, Serrano J, Gomez-Espí M, Rodriguez M, Del Vale E, Gracia-Sabrido JL, Ferrer C, Sole C (2014). Interval between neoadjuvant treatment and definitive surgery in locally advanced rectal cancer: impact on response and oncologic outcomes. Journal of Cancer Research and Clinical Oncology.

[ref-2] Du D, Su Z, Wang D, Liu W, Wei Z (2018). Optimal interval to surgery after neoadjuvant chemoradiotherapy in rectal cancer: a systematic review and meta-analysis. Clinical Colorectal Cancer.

[ref-3] Francois Y, Nemoz CJ, Baulieux J, Vignal J, Grandjean J-P, Partensky C, Souquet JC, Adeleine P, Gerard J-P (1999). Influence of the interval between preoperative radiation therapy and surgery on downstaging and on the rate of sphincter-sparing surgery for rectal cancer: the lyon R90-01 randomized trial. Journal of Clinical Oncology Official Journal of the American Society of Clinical Oncology.

[ref-4] Garcia-Aguilar J, Chow OS, Smith DD, Marcet JE, Cataldo PA, Varma MG, Kumar AS, Oommen S, Coutsoftides T, Hunt SR, Stamos MJ, Ternent CA, Herzig DO, Fichera A, Polite BN, Dietz DW, Patil S, Avila K (2015). Effect of adding mFOLFOX6 after neoadjuvant chemoradiation in locally advanced rectal cancer: a multicentre, phase 2 trial. Lancet Oncology.

[ref-5] Habr-Gama A, Perez RO, Nadalin W, Sabbaga J, Gama-Rodrigues J (2004). Operative versus nonoperative treatment for stage 0 distal rectal cancer following chemoradiation therapy long-term results. Annals of Surgery.

[ref-6] Kalady MF, De CamposLobato LF, Stocchi L, Geisler DP, Dietz D, Lavery IC, Fazio VW (2015). Predictive factors of pathologic complete response after neoadjuvant chemoradiation for rectal cancer. Annals of Surgery.

[ref-7] Kerr SF, Norton S, Glynne-Jones R (2008). Delaying surgery after neoadjuvant chemoradiotherapy for rectal cancer may reduce postoperative morbidity without compromising prognosis. British Journal of Surgery.

[ref-8] Kim SY, Joo J, Kim TW, Hong YS, Kim JE, Hwang IG, Kim BG, Lee K-W, Kim J-W, Oh H-S, Ahn JB, Zang DY, Kim DY, Oh JH, Baek JY (2018). A randomized phase 2 trial of consolidation chemotherapy after preoperative chemoradiation therapy versus chemoradiation therapy alone for locally advanced rectal cancer: KCSG CO 14-03. International Journal of Radiation Oncology*Biology*Physics.

[ref-9] Ludmir EB, Palta M, Willett CG, Czito BG (2017). Total neoadjuvant therapy for rectal cancer: an emerging option. Cancer.

[ref-10] Maas M, Nelemans PJ, Valentini V, Das P, Rödel C, Kuo L-J, Calvo FA, García-Aguilar J, Glynne-Jones R, Haustermans K, Mohiuddin M, Pucciarelli S, Small W, Suárez J, Theodoropoulos G, Biondo S, Beets-Tan RGH, Beets GL (2010). Long-term outcome in patients with a pathological complete response after chemoradiation for rectal cancer: a pooled analysis of individual patient data. Lancet Oncology.

[ref-11] Macfarlane JK, Ryall RD, Heald RJ (1993). Mesorectal excision for rectal cancer. British Journal of Surgery.

[ref-12] Marco MR, Zhou L, Patil S, Marcet JE, Varma MG, Oommen S, Cataldo PA, Hunt SR, Kumar A, Herzig DO, Fichera A, Polite BN, Hyman NH, Ternent CA, Stamos MJ, Pigazzi A, Dietz D, Yakunina Y, Pelossof R, Garcia-Aguilar J (2018). Consolidation mFOLFOX6 chemotherapy after chemoradiotherapy improves survival in patients with locally advanced rectal cancer: final results of a multicenter phase II trial. Diseases of the Colon & Rectum.

[ref-13] Moore HG, Gittleman AE, Minsky BD, Wong D, Paty PB, Weiser M, Temple L, Saltz L, Shia J, Guillem JG (2004). Rate of pathologic complete response with increased interval between preoperative combined modality therapy and rectal cancer resection. Diseases of the Colon & Rectum.

[ref-14] Roh MS, Colangelo LH, O’Connell MJ, Yothers G, Deutsch M, Allegra CJ, Kahlenberg MS, Baez-Diaz L, Ursiny CS, Petrelli NJ, Wolmark N (2009). Preoperative multimodality therapy improves disease-free survival in patients with carcinoma of the rectum: NSABP R-03. Journal of Clinical Oncology.

[ref-15] Seegenschmiedt MH (2004). Preoperative versus postoperative chemoradiotherapy for rectal cancer. Journal of clinical oncology.

[ref-16] Sloothaak DAM, Geijsen DE, Van Leersum NJ, Punt CJA, Buskens CJ, Bemelman WA, Tanis PJ (2013). Optimal time interval between neoadjuvant chemoradiotherapy and surgery for rectal cancer. British Journal of Surgery.

[ref-17] Smith JJ, Chow OS, Gollub MJ, Nash GM, Temple LK, Weiser MR, Guillem JG, Paty PB, Avila K, Garcia-Aguilar J, on behalf of the Rectal Cancer Consortium (2015). Organ preservation in rectal adenocarcinoma: a phase II randomized controlled trial evaluating 3-year disease-free survival in patients with locally advanced rectal cancer treated with chemoradiation plus induction or consolidation chemotherapy, and total mesorectal excision or nonoperative management. BMC Cancer.

[ref-18] Smith JD, Ruby JA, Goodman KA, Saltz LB, Guillem JG, Weiser MR, Temple LK, Nash GM, Paty PB (2012). Nonoperative management of rectal cancer with complete clinical response after neoadjuvant therapy. Annals of Surgery.

[ref-19] Tulchinsky H, Shmueli E, Figer A, Klausner JM, Rabau M (2008). An interval >7 weeks between neoadjuvant therapy and surgery improves pathologic complete response and disease-free survival in patients with locally advanced rectal cancer. Annals of Surgical Oncology.

[ref-20] Van der Valk MJM, Hilling DE, Bastiaannet E, Meershoek-Klein Kranenbarg E, Beets GL, Figueiredo NL, Habr-Gama A, Perez RO, Renehan AG, Van de Velde CJH, Ahlberg M, Appelt A, Asoglu O, Bär M-T, Barroca R, Beets-Tan RGH, Belgers EHJ, Bosker RJI, Breukink SO, Bujko K, Carvalho C, Cunningham C, Creavin B, D’Hoore A, Gérard J-P, Gollins S, Hoff C, Holman FA, Hupkens BJP, Iseas S, Jakobsen A, Keshvari A, Koopal SA, Kusters M, Langheinrich M, Leijtens JWA, Maas M, Malcomson L, Mamedli ZZ, Martling A, Matzel KE, Melenhorst J, Morici ML, Murad-Regadas SM, O’Dwyer ST, Peeters KCMJ, Rosa I, Rossi G, Rutten HJT, Sanchez Loria F, Van der Sande ME, São Julião GP, Saunders M, Sun Myint A, Van der Sluis H, Schiappa R, Scott N, Stoot JHMB, Talsma AK, Terrasson I, Tokmak H, Vaccaro CA, Vahrmeijer AL, Wasowicz DK, Westreenen HL, Winter DC, Wolthuis AM, Zimmerman DDE (2018). Long-term outcomes of clinical complete responders after neoadjuvant treatment for rectal cancer in the international watch & wait database (IWWD): an international multicentre registry study. Lancet.

[ref-21] Wolthuis AM, Penninckx F, Haustermans K, De Hertogh G, Fieuws S, Van Cutsem E, D’Hoore A (2012). Impact of interval between neoadjuvant chemoradiotherapy and TME for locally advanced rectal cancer on pathologic response and oncologic outcome. Annals of Surgical Oncology.

[ref-22] Zeng WG, Zhou Z-X, Liang J-W, Wang Z, Hou H-R, Zhou H-T, Zhang X-M, Hu J-J (2014). Impact of interval between neoadjuvant chemoradiotherapy and surgery for rectal cancer on surgical and oncologic outcome. Journal of Surgical Oncology.

[ref-23] Zorcolo L, Rosman AS, Restivo A, Pisano M, Nigri GR, Fancellu A, Melis M (2012). Complete pathologic response after combined modality treatment for rectal cancer and long-term survival: a meta-analysis. Annals of Surgical Oncology.

